# Chromosome 1q21 translocation and spermatogenesis failure: Two case reports and review of the literature

**DOI:** 10.1097/MD.0000000000018588

**Published:** 2019-12-27

**Authors:** Ranwei Li, Xiuyang Wang, Shuqiang Feng, Xiao Yang, Qiushuang Zhang, Peng Zhan

**Affiliations:** Department of Urology, the Second Hospital of Jilin University, Changchun, China.

**Keywords:** azoospermia, breakpoint, chromosome 1, genetic counseling

## Abstract

**Rationale:**

For the carriers of chromosome reciprocal translocation, the reason why some are fertile and others are infertile remains unclear. Here, we describe 2 patients who are carriers of chromosome 1q21 translocation with azoospermia.

**Patient concerns:**

A 29-year-old male and a 33-year-old male presented at the clinic with a diagnosis of infertility.

**Diagnosis:**

Both patients with azoospermia were diagnosed with Routine semen analysis, cytogenetic diagnosis and detection of serum reproductive hormones. The karyotype results of 2 patients were 46,XY,t(1;17)(q21;q23) and 46,XY,t(1;10)(q21;p12), respectively.

**Interventions:**

After genetic counseling and informed consent, 1 patient (Case 2) chose microsopic testicular sperm extraction (micro-TESE).

**Outcomes:**

After micro-TESE, no sperm was found for the patient. Finally, both patients chose clinical treatment through artificial insemination with donor sperm.

**Lessons:**

These outcomes suggest that breakpoint at 1q21 should be paid attention by physician in genetic counseling, may harbor some genes associated with spermatogenesis, and deserves further be studied on the function of related genes.

## Abstract 2

Supplemental Digital Content is available in the text

## 1 Introduction

Infertility affects approximately 22% of couples in reproductive age,^[1]^ and about 50 million couples worldwide.^[2]^ Male infertility constitutes 50% of these couples,^[3]^ affects approximately 4% of all men worldwide.^[4]^ Several studies have shown that male infertility is attributed to multiple causes, mainly due to a failure in spermatogenesis.^[3]^ The spermatogenic failure directly results in azoospermia or severe oligozoospermia.^[5]^ Although the cause of the severe cases often remains unknown, genetic factors can lead to spermatogenic impairment. Chromosomal abnormalities or microdeletions of the AZF region on Y chromosome can disrupt spermatogenesis.^[6]^

Reciprocal chromosome translocations are one of the main genetic factors for the male carriers with reproductive failures. The certain translocations or chromosomal breakpoints directly disrupt spermatogenesis, and result in abnormal sperm concentration.^[7]^ Chromosome breakpoints involved in translocations has been paid attention to in recent years.[^8^,^9^,^10^] Paoloni-Giacobino et al^[11]^ reported a familial t(6;21)(p21.1;p13) translocation is associated with male-only sterility, and male carrier showed abnormal spermatocyte meiosis. Ananthapur et al^[1]^ reported a male carrier with de novo chromosomal translocation t(2;11)(p14;q21), and the translocation may result in the disruption of genes responsible for spermatogenesis. If translocation breakpoints interrupt a vital gene structures, the carrier likely suffer spermatogenic failure.

Previous studies have shown that chromosome 1 could harbor an important domain whose integrity is very important for spermatogenesis, and that chromosome 1q21 is the largest number of breakpoints.^[12]^ This study was established to identify 2 male cases of chromosome 1q21 translocation. Combining published cases, this paper also discuss the association between this breakpoint and spermatogenesis.

## 2 Case reports

This study included 2 male carriers with chromosome 1q21 translocation, which showed azoospermia. Approval of this study was obtained from the Ethics Committee of the Second Hospital, Jilin University (No. 2019-032). Patients have provided informed consent for publication of 2 cases.

### 2.1 Case 1

**Figure 1:**
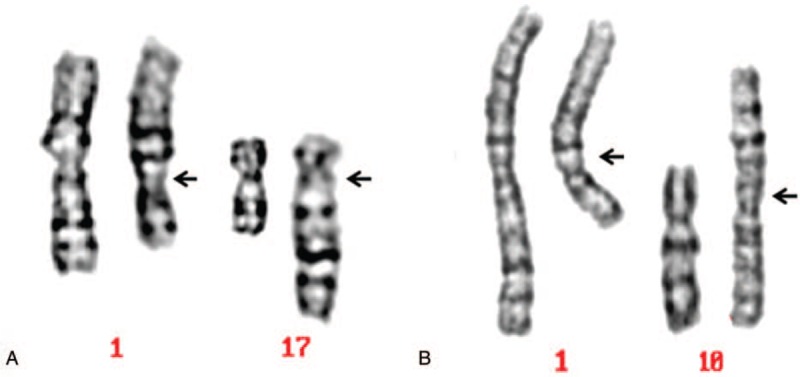
Abnormal karyotypes of possessing chromosome 1q21 translocations.

A 29-year-old man presented at the clinic with a diagnosis of infertility. The patient had normal appearance and intelligence, and went to the andrology outpatient department because of being childless after 5 years of marriage. He was subjected to 2 routine semen analyses, which were 2 weeks interval. No sperm was found twice. Then the patient underwent serum reproductive hormone and cytogenetic detection. The results of reproductive hormones were as follows: FSH: 25.8U/L, LH: 8.6 U/L, T: 18.6 nmol/L (Normal reference value of serum reproductive hormones: FSH: 1.5–12.4 U/L; LH: 1.7–8.6 U/L; T: 9.9–27.8 nmol/L). G band karyotype analysis was 46,XY,t(1;17)(q21;q23) (Fig. 1A).

### 2.2 Case 2

A 33-year-old man presented at the clinic with a diagnosis of infertility. The patient had normal appearance and intelligence, and went to the andrology outpatient department experiencing infertility after 10 years of marriage. No sperm was found to undergo 2 semen analyses at intervals of 2 weeks. The detection of reproductive hormone indicated that E_2_ and T were both lower than normal reference value (FSH: 10.6 U/L, LH: 7.5 U/L, T: 1.63 nmol/L). The results of cytogenetic was 46,XY,t(1;10)(q21;p12) (Fig. 1B). After genetic counseling and informed consent, the patient chose microsopic testicular sperm extraction. Unfortunately, no sperm was found.

### 2.3 Literature review

A search for reports on chromosome 1q21 translocations from infertile men was performed using PubMed. The keywords were “chromosome 1/ translocation / infertility / male” for the search. The cases of chromosomal 1 translocation were collected and classified. We included cases of chromosome 1q21 translocation for adult fertile-age men, and excluded 2 cases without clinical manifestation. A total of 19 carriers involving chromosomal 1q21 translocation were found. Karyotype and clinical findings involved chromosome 1q21 breakpoints from literature analysis are shown in Table 1. The results showed that 94.7% (18/19) of the cases presented with spermatogenesis disorder.

**Table 1:**
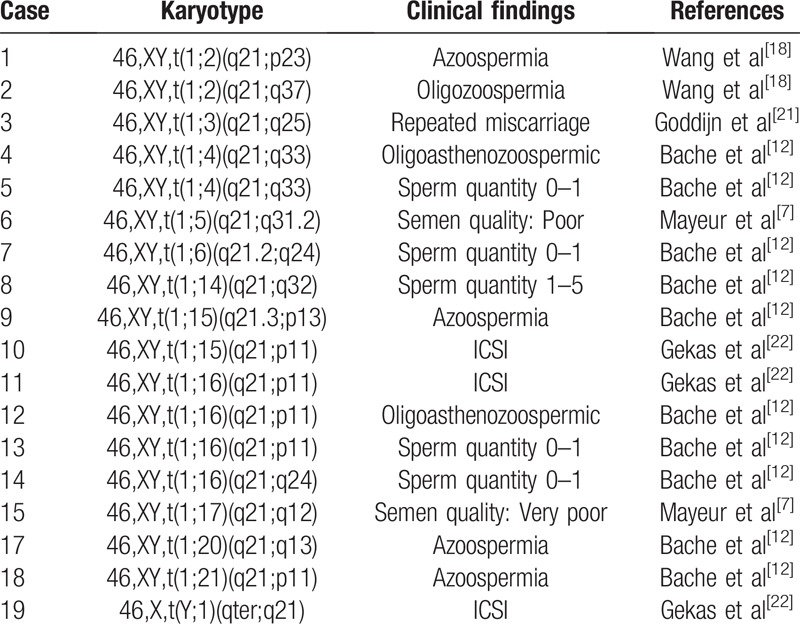
Chromosome 1q21 breakpoints in translocation carriers reported in previous literature.

## 3 Discussion

In this study, we report 2 cases of chromosome 1q21 translocation in infertile men who are azoospermic patients. One case presented with an increased level of FSH, and the other case was a decrease in serum testosterone level. Previous studies have confirmed that FSH is essential for the regulation of spermatogenesis, and also T plays an important role.^[13]^ Dong et al^[14]^ once reported that chromosomal translocations may cause reductions in testosterone level. Similarly, Uccellatore et al^[15]^ speculated that some translocation may be accompanied by reproductive hormone disorders. But, the exact relationship between chromosome translocation and reproductive hormone disorders is not clear. The special mechanism deserves further study.

For the carriers of chromosome reciprocal translocation, the reason why some are fertile and others are infertile remains unclear. It might be speculated that the specific chromosomes and breakpoints are involved in the translocation, and some breakpoints can disrupt the structure of an important gene, leading to spermatogenic disorders.^[16]^ Some studies have suggested that chromosome 1 may harbor a critical domain, which are essential for male fertility.[^12^,^17^] Bache et al^[12]^ reported that breakpoint at 1q21 is the largest number reported in male infertility patients. Wang et al^[18]^ reported that the breakpoint at 1q21 were associated with pre-gestational infertility, which characterized by failure to fertilize eggs. Chromosome 1q21 translocation was involved in our 2 cases. The breakpoint at 1q21 involving translocation was searched and analyzed by PubMed. Of the 19 carriers, 94.7% had spermatogenesis disorder. This suggests that specific chromosome breakpoint should be paid attention by physician in genetic counseling.

By OMIM search, we found 17 genes expressed in testis, located the breakpoint on chromosome 1q21. List of genes located on chromosome 1q21 were collected and summarized in a supplementary file (Table S1). The function of these genes in testis is not clear. Of the 17 genes, horma domain-containing 1 (HORMAD1) gene is located on chromosome 1q21.3, and its expression in testis coincided with the onset of meiosis I;^[19]^ Ornithine decarboxylase antizyme 3 (OAZ3) gene, mapped on chromosome 1 at 1q21.3, began to express in the early stage of spermatogenesis and ended in the late spermatid phase.^[20]^ The relationship between these genes and azoospermia needs further study.

A limitation of this study is the lack of the research on the function of the genes involving breakpoint. Therefore, we are unable to confirm whether the genetic structure associated with spermatogenesis has changed. The suggested detection of gene function needs to be validated in more cases.

In conclusion, this study reported 2 carriers of chromosome 1q21 translocation with azoospermia. The breakpoint should be paid attention by physician in genetic counseling. Breakpoint at 1q21 may harbor some genes associated with spermatogenesis, deserves further be studied on the function of related genes.

## Author contributions

**Conceptualization:** Ranwei Li.

**Investigation:** Shuqiang Feng, Xiao Yang.

**Methodology:** Qiushuang Zhang, Peng Zhan.

**Writing – original draft:** Xiuyang Wang.

**Writing – review & editing:** Ranwei Li.

Ranwei Li orcid: 0000-0002-5556-6285

## Supplementary Material

Supplemental Digital Content
