# Supplementary material for: Chromosome 1q21 translocation and spermatogenesis failure: Two case reports and review of the literature
Source: Medicine (Baltimore). 2019 Dec 27;98(52):e18588. doi: 10.1097/MD.0000000000018588 (PMC6946535; doi:10.1097/MD.0000000000018588)
Supplement: Supplemental Digital Content [file medi-98-e18588-s001.docx]

Supplementary data

Table S1. List of genes located on chromosome 1q21

| Gene | Cytogenetic locations | Expressed in testis | Function in testis | Reference |
| --- | --- | --- | --- | --- |
| HYDIN2 | 1q21.1 | Lower expression | Unknown | Nagase et al. ^[23]^ |
| NBPF12 | 1q21.1 | Expression | Unknown | Vandepoele et al. ^[24]^ |
| ADAMTSL4 | 1q21.2 | Expression | Unknown | Ahram et al.^[25]^ |
| ACP6 | 1q21.2 | Higher expression | Unknown | Takayama et al. ^[26]^ |
| NBPF9 | 1q21.2 | Expression | Unknown | Vandepoele et al. ^[27]^ |
| PRPF3 | 1q21.2 | Expression | Unknown | Tanackovic et al.^[28]^ |
| SV2A | 1q21.2 | Intermediate expression | Unknown | Nagase et al.^[29]^ |
| VPS45 | 1q21.2 | Abundant expression | Unknown | Pevsner et al.^[30]^ ;  Rajasekariah et al.^[31]^ |
| CREB3L4 | 1q21.3 | Expression | Unknown | Cao et al.^[32]^ |
| EFNA3 | 1q21.3 | Expression | Unknown | Kozlosky et al.^[33]^ |
| [FLG2](https://www.ncbi.nlm.nih.gov/omim/616284) | 1q21.3 | Expression | Unknown | Wu et al.^[34]^ |
| HORMAD1 | 1q21.3 | High expression | Its expression in testis coincided with the onset of meiosis I. | Pangas et al.^[19]^ |
| OAZ3 | 1q21.3 | 1.1-kb OAZ3 transcript in testis only | Expression starts early in spermiogenesis and finishes in the late spermatid phase. | Ivanov et al.^[20]^ |
| SMCP | 1q21.3 | Abundant expression | The maintenance and stabilization of the crescent structure of the sperm mitochondria | Hawthorne et al.^[35]^ |
| THEM4 | 1q21.3 | Highest expression | Unknown | Maira et al.^[36]^ |
| ERVK-7 | 1q21-q23 | Expression | Unknown | Sugimoto et al.^[37]^ |

**References**

[23]Nagase T, Nakayama M, Nakajima D, et al. Prediction of the coding sequences of unidentified human genes. XX. The complete sequences of 100 new cDNA clones from brain which code for large proteins in vitro. DNA Res 2001; 8: 85-95.

[24]Vandepoele K, Van Roy N, Staes K, et al. A novel gene family NBPF: intricate structure generated by gene duplications during primate evolution. Molec Biol Evol 2005; 22: 2265-74.

[25]Ahram D, Sato TS, Kohilan A, et al. A homozygous mutation in ADAMTSL4 causes autosomal-recessive isolated ectopia lentis. Am J Hum Genet 2009; 84: 274-8.

[26]Takayama I, Daigo Y, Ward SM, et al. Novel human and mouse genes encoding an acid phosphatase family member and its downregulation in W/Wv mouse jejunum. Gut 2002; 50: 790-6.

[27]Vandepoele K, Van Roy N, Staes K, et al. A novel gene family NBPF: intricate structure generated by gene duplications during primate evolution. Mol Biol Evol 2005; 22:2265-74.

[28] Tanackovic G, Ransijn A, Thibault P, et al. PRPF mutations are associated with generalized defects in spliceosome formation and pre-mRNA splicing in patients with retinitis pigmentosa. Hum Mol Genet 2011; 20: 2116-30.

[29]Nagase T, Ishikawa K, Suyama M, et al. Prediction of the coding sequences of unidentified human genes. XI. The complete sequences of 100 new cDNA clones from brain which code for large proteins in vitro. DNA Res 1998; 5:277-86.

[30]Pevsner J, Hsu SC, Hyde PS, et al. Mammalian homologues of yeast vacuolar protein sorting (vps) genes implicated in Golgi-to-lysosome trafficking. Gene 1996; 183:7-14.

[31] Rajasekariah P, Eyre HJ, Stanley KK, et al. Molecular cloning and characterization of a cDNA encoding the human leucocyte vacuolar protein sorting (hlVps45). Int J Biochem Cell Biol 1999;31: 683-94.

[32]Cao G, Ni X, Jiang M, et al. Molecular cloning and characterization of a novel human cAMP response element-binding (CREB) gene (CREB4). J Hum Genet 2002; 47: 373-6.

[33] Kozlosky CJ, Maraskovsky E, McGrew JT, et al. Ligands for the receptor tyrosine kinases hek and elk: isolation of cDNAs encoding a family of proteins. Oncogene 1995;10: 299-306.

[34]Wu Z, Hansmann B, Meyer-Hoffert U, et al. Molecular identification and expression analysis of filaggrin-2, a member of the S100 fused-type protein family. PLoS One 2009; 4: e5227.

[35] Hawthorne SK, Goodarzi G, Bagarova J, et al. Comparative genomics of the sperm mitochondria-associated cysteine-rich protein gene. Genomics 2006; 87: 382-91.

[36]Maira SM, Galetic I, Brazil DP, et al. Carboxyl-terminal modulator protein (CTMP), a negative regulator of PKB/Akt and v-Akt at the plasma membrane. Science 2001; 294: 374-80.

[37]Sugimoto J, Matsuura N, Kinjo Y, et al. Transcriptionally active HERV-K genes: identification, isolation, and chromosomal mapping. Genomics 2001; 72: 137-44.
